# *T*. *vaginalis* Infection Is Associated with Increased IL-8 and TNFr1 Levels but with the Absence of CD38 and HLADR Activation in the Cervix of ESN

**DOI:** 10.1371/journal.pone.0130146

**Published:** 2015-06-17

**Authors:** Olamide D. Jarrett, Kirsten E. Brady, Sharada P. Modur, Jill Plants, Alan L. Landay, Mahmood Ghassemi, Elizabeth T. Golub, Greg T. Spear, Richard M. Novak

**Affiliations:** 1 Section of Infectious Diseases, Department of Medicine, University of Illinois at Chicago School of Medicine, Chicago, Illinois, 60612, United States of America; 2 Department of Immunology/Microbiology, Rush University Medical Center, Chicago, Illinois, 60612, United States of America; 3 Department of Epidemiology, Johns Hopkins Bloomberg School of Public Health, Baltimore, Maryland, 21205, United States of America; University of Cape Town, SOUTH AFRICA

## Abstract

**Introduction:**

*Trichomonas vaginalis* infection is associated with an increased risk of HIV infection in exposed-seronegative women (ESN) despite their unique immune quiescent profile. It is important to understand possible mechanisms, such as recruitment of activated T cells, by which *T*. *vaginalis* could facilitate HIV infection in this population.

**Methods:**

We conducted a cross-sectional study exploring the relationships between *T*. *vaginalis* infection, inflammatory markers and T cell activation in the cervix of ESN. During scheduled study visits, participants completed a behavioral questionnaire and physical exam, including sexually transmitted infection (STI) screening and collection of endocervical sponge and cytobrush specimens. T cell and monocyte phenotypes were measured in cervical cytobrush specimens using multi-parameter flow cytometry. Cervical sponge specimens were used to measure cytokines (IL-6, IL-8,IL-10, IP-10, RANTES) using Luminex immunoassays and the immune activation marker soluble TNF receptor 1 using ELISA.

**Results:**

Specimens of 65 women were tested. Twenty-one of these women were infected with *T*. *vaginalis*. *T*. *vaginalis* infection was associated with significantly increased concentrations of IL-8 (1275pg/ml vs. 566pg/ml, p=.02) and sTNFr1 (430 pg/ml vs. 264 pg/ml, p=.005). However, *T*. *vaginalis* infection was not associated with increased percent expression of CCR5+ T cells nor increased CD38 and HLADR activation compared to uninfected women. It was also not associated with increased expression of CCR5+ monocytes.

**Conclusions:**

Among ESN *T*. *vaginalis* infection is associated with increased levels of genital pro-inflammatory/immune activation markers IL-8 and TNFr1, but was not associated with an increased percentage of activated endocervical T cells along the CD38 and HLADR pathways. Thus, while *T*.*vaginalis* infection may result in some reversal of the immune quiescent profile of ESN, enhanced recruitment of activated CD38 and HLADR expressing CD4+ cells into the endocervix may not be part of the mechanism by which *Trichomonas* infection alters HIV susceptibility in this unique subset of women.

## Introduction

*Trichomonas vaginalis* is the most prevalent treatable sexually transmitted infection (STI), with an estimated 135 million cases in women world-wide, and has been associated with a 1.5–2.7 fold increased risk of HIV-1 infection among exposed seronegative women (ESN), including female sex workers [1–5]. However, the mechanisms by which *T*. *vaginalis* confers an increased risk of HIV infection are still unclear.

Multiple studies suggest that sexual transmission of HIV infection in women occurs preferentially in the setting of immune activation/inflammation. HIV has been found to preferentially infect CCR5 expressing and CD38+CD4+ T cells in the cervix [6]. Similarly, increased HIV viral replication in the female genital tract has been associated with increased levels of pro-inflammatory cytokines such as interleukin (IL)-8, IL-6, interferon gamma-induced protein (IP)-10 and tumor necrosis factor alpha (TNF-a) in the genital tract [7–11]. While the predominance of evidence supports that HIV transmission is facilitated by immune activation/inflammation, it is not a consistent association. RANTES, a pro-inflammatory cytokine, has been associated with decreased HIV viral replication.[12] Increased levels of IL-10, an anti-inflammatory cytokine, have been associated with both stimulation and suppression of HIV viral replication in the female genital tract [13, 14].

Lajoie et al provide further support for the potential role of immune activation in sexual transmission of HIV disease. They found that ESN have a uniquely immune quiescent profile with lower levels of a broad range of cytokines, including IL-6, IL-10, interferon gamma-induced protein (IP)-10 and TNF- in the genital tract of Nairobi sex workers compared to women at low risk of HIV infection [15]. In addition, they found that ESN had a higher proportion of CD4+ cells compared to low risk women but a reduced percentage of CD4+ cells expressing the CCR5+ phenotype. This immune quiescent profile may help explain ESN resistance to HIV acquisition despite sexual exposure.

In light of these results, it is of interest to explore whether *T*. *vaginalis* infection may stimulate immune activation in the female genital tract, reversing the immune quiescent state of ESN, and potentially leading to an increased risk of HIV transmission. Multiple studies have demonstrated that *T*. *vaginalis* infection is associated with increased expression of IL-8, IL-6 and TNF-a [8, 12, 16–19]. In contrast, there is limited data about the impact of *Trichomonas* infection on other cytokines and markers of immune activation such RANTES, IL-10 and interferon gamma-induced protein IP-10. Henning et al found that co-infection of *Chlamydia trachomatis* and *T*. *vaginalis* was associated with decreased levels of RANTES and increased HIV infectivity in a macaque model, but it is unknown if these associations hold true in the setting of *T*. *vaginalis* mono-infection [12].

There is also limited data on the impact of *T*. *vaginalis* infection on the presence of T cells and monocytes. Guenther et al demonstrated *in vitro* that co-culture of *T*. *vaginalis* with peripheral blood mononuclear cells (PBMC) induced an activated phenotype with increased CD69+ and HLA-DR expression.[16] However no studies to date have investigated the association between *T*. *vaginalis* infection and phenotypic expression of T cells and monocytes in the female genital tract.

Thus, we investigated whether *T*. *vaginalis* infection in the genital tract of ESN is associated with increased percentage of T cells, including those with an activated phenotype, and monocytes in endocervical tissue. We also explored whether this occurred in the setting of increased genital levels of cytokines/chemokines associated with stimulation and inhibition of HIV infection and replication: IL-6, IL-8, IP-10, IL-10, and RANTES. We also measured soluble TNF-a receptor 1 (sTNFr1) as a proxy for TNF-a induced immune activation based on prior work by Spear et al demonstrating largely undetectable levels of TNF-a in cervico-vaginal lavage specimens [10].

## Methods

### Subjects

We conducted a cross-sectional analysis of 65 ESN enrolled in a University of Illinois at Chicago study exploring factors related to genital mucosal immunity against HIV infection. Women in this cohort were between the ages of 18–45 and reported engaging in at least 2 of the following criteria: exchange of sex for money, drugs or shelter in the last 6 months; at least 5 sexual partners in the last 6 months, history of a sexually transmitted infection (STI) in the last year, and/or sexual relations with a known HIV positive man. All the women enrolled in the study reported that they were actively engaged in sex work.

### Ethics statement

This study was approved by the Institutional Review Boards of the University of Illinois at Chicago and Rush Medical Center. All women enrolled in the study provided written informed consent.

### Study protocol and sample collection

The ESN enrolled in the study presented for scheduled study visits every 6 months during which they completed behavioral questionnaires and physical exams, including pelvic exam for collection of cervical and vaginal samples, as has been previously described [20]. Last menstrual period was recorded each visit and women were characterized as being in the luteal phase or follicular phase based on a 28-day menstrual cycle. Women who reported being menopausal were recorded as such.

Endocervical sponge and cytobrush samples were collected for each participant on the same visit and were used to measure cytokine/immune marker concentrations and immune cell phenotypes, respectively. Sponge samples were collected prior to cytobrush samples. After sample collection, the sponge was weighed to determine volume then processed in 600 microliters of PBS and 60microliters of 10% Aprotinin. Sponge samples were then stored at -70°C in 100 microliter aliquots prior to testing. Cytobrush specimens were spun and processed within two hours of sample collection to maximize yield of viable T cells extracted from the specimens. Specimens used in our study were collected during the participants’ first or second study visit.

### Testing procedures

HIV testing at each visit was performed using Oraquick (OraSure Technologies, Inc). *T*. *vaginalis* infection was determined from vaginal sampling using the OSOM Trich assay (Genzyme Diagnostics, Cambridge, MA), an immunochromatographic capillary-flow enzyme immunoassay dipstick test [21]. Bacterial vaginosis was determined using Nugent’s criteria [22]. *Chlamydia trachomatis* and *Neisseria gonorrhea* testing were performed using APTIMA Combo 2 Assay (GenProbe and Hologic, Inc).

Luminex multiplex immunoassays (Bio-Plex Pro Human Chemokine Assays, Bio-Rad, Inc) were used to quantify the following cytokines in the cervical sponge samples: IL-6, IL-8, IL-10, IP-10, and RANTES. The lower limits of detection (LLD) for these assays were: IL-6 0.65 pg/ml; IL-8 0.49 pg/ml; IL-10 1.20 pg/ml; IP-10 0.84 pg/ml; RANTES 5.75 pg/ml. Soluble TNFr1 was measured using a commercial ELISA kit by R&D systems (Catalog number DRT100) with LLD of 7.8 pg/ml. Samples with values below the LLD for any given assay were recoded to a value equal to that assay LLD/√2 [23].

T cell and monocyte phenotypes were measured in cytobrush specimens using multi-parameter flow cytometry and the gating strategy used was performed according to the Mucosal Immunology Group protocol [24]. The following fluorochrome-conjugated antibodies were used: anti-CD3, anti-CD8, anti-CD45, anti-CD38, anti-HLA-DR, anti-CCR5 (all Becton Dickenson); anti-CD4 (Invitrogen); and anti-CD14 (Beckman Coulter)[25, 26]. Antibodies were used at the manufacturer recommended dilutions. Anti-CD38 and anti-HLA-DR were used to determine T cell activation [25]. Anti-CCR5 was used to study the association between *T*. *vaginalis* infection and HIV entry receptor CCR5 expression in T cells and monocytes. Flow cytometry analysis was performed using FlowJo software (Treestar).

### Statistical analysis

Statistical analysis was performed using SAS v. 9.3 (SAS Institute, Inc), R v. 2.15.2 (GNU Project), and GraphPad Prism (version 6.0, GraphPad Software, La Jolla, CA). Distributions of each outcome were explored to assess for normality. For baseline characteristics, Mann-Whitney test was used for continuous outcomes and Chi-squared or Fisher exact (when >20% of cells with expected count <5) tests were used for discrete variables. The associations between cytokines, sTNFr1 and *T*. *vaginalis* infection were explored using Mann-Whitney test. Beta regression was used to model the relationship between T cell and monocyte phenotypes and *T*. *vaginalis* infection. Statistical significance was defined as p<.05.

## Results

### Sociodemographics

The median age of women studied was 42 with no significant association between age and *T*. *vaginalis* infection (Table 1). Surprisingly, while women in this study met criteria for sexual exposure to HIV infection, they reported a low number of sexual encounters with median of one episode of unprotected sex in the week prior to the study visit and only one sexual partner in the 6 months prior to the study visit. There was a significant difference (p = .03) in menstrual phase between the two groups with all menopausal women (N = 12) in the uninfected group. There was a trend toward higher contraception use in the *T*. *vaginalis* infected group (95% vs. 71%, p = 0.05), with condoms being the primary mode of contraception used in both groups. There was a similar prevalence of bacterial vaginosis in *T*. *vaginalis* infected versus uninfected women (10% versus 9%, p = 1.00) and a low prevalence of co-infection with other STIs, with no significant difference between groups (Chlamydia, p = 0.55; *Neisseria Gonorrhea*, p = 1.00) Of note, none of the *T*. *vaginalis* infected women reported experiencing any unusual/abnormal vaginal symptoms at the time of their study visit when asked if they had any vaginal/genital complaints. Therefore, the *T*. *vaginalis* infections in this study are presumed to be asymptomatic infections.

**Table 1:** Baseline characteristics of ESN by *T*. *vaginalis* status.

	TV infected (n = 21)	TV uninfected (n = 44)	p-value
Median age (IQR)	42 (39,45)	42.5 (39,47)	.67
Median episodes unprotected sex, last 7 days (IQR)	1 (1,2)	1 (0,2.5)	.19
Median unprotected sexual partners, last 6 months (IQR)	1 (1,2)	1 (1,5.5)	.32
Any contraception	20 (95)	32 (73)	.05
Condom only	13 (62)	22 (50)	.37
Condom + IUD/tubal	6 (29)	6 (13)	.18
IUD or tubal	1 (5)	4 (9)	1.00
Bacterial vaginosis	2 (10)	4 (9)	1.00
*Neisseria Gonorrhea*	0	1 (2)	1.00
Chlamydia	0	3 (7)	.55
Menstrual phase			.03
Luteal phase	8 (38)	14 (32)	
Follicular phase	13 (62)	18 (41)	
Menopause	0	12 (27)	

ESN = female sex workers, TV = *T*. *vaginalis*, IUD = intrauterine device, tubal = tubal ligation, STI = sexually transmitted infection; Values represent N (%) unless otherwise noted.

### Endocervical Cytokine and sTNFr1 levels

**Fig 1:**
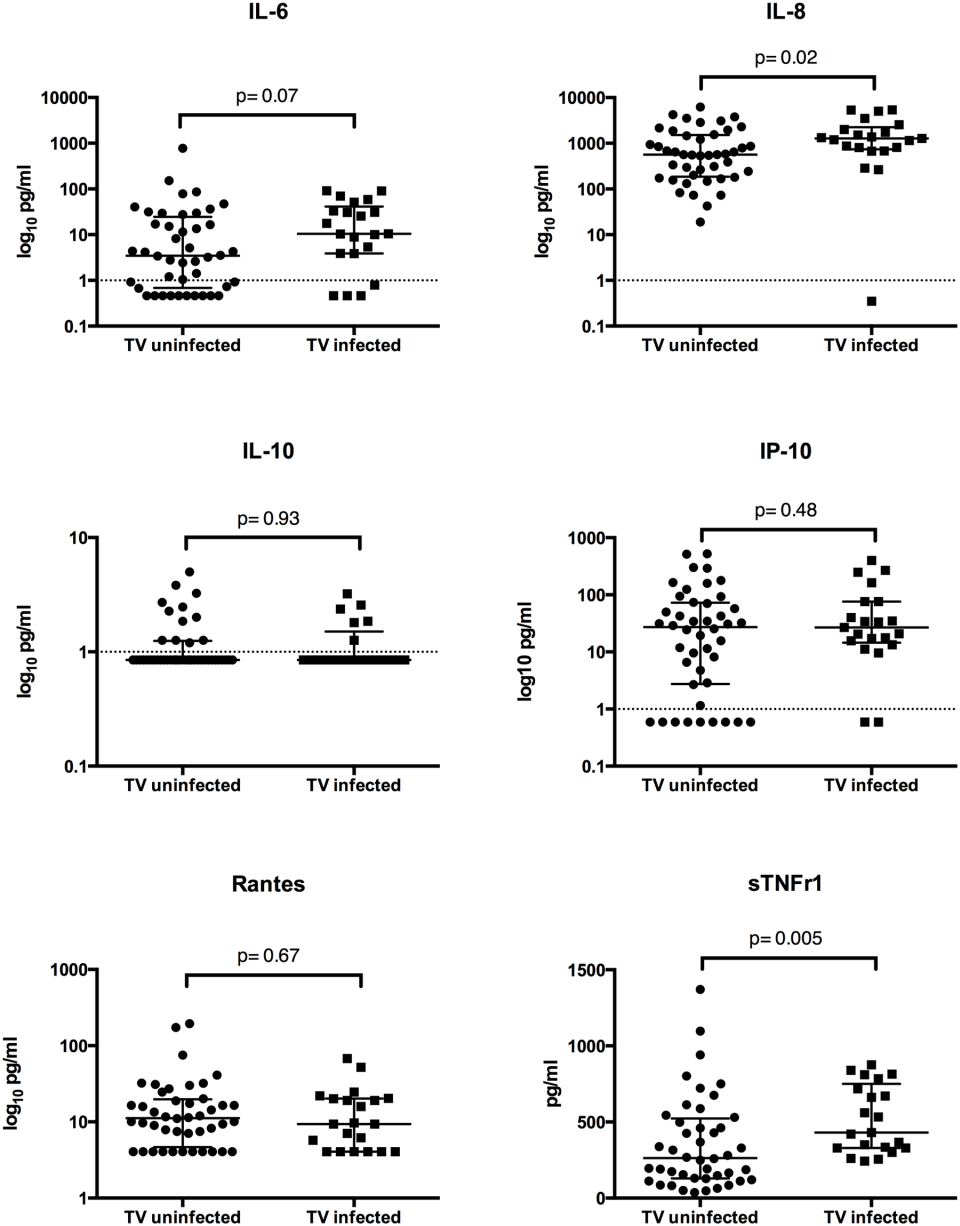
Cytokines and sTNFr1 endocervical expression. Data represents median and interquartile range. TV = *Trichomonas vaginalis*. P values are the result of Mann-Whitney test.

Fig 1 shows the relationship between endocervical cytokines/inflammatory markers and *T*. *vaginalis* infection. With the exception of IL-8, cytokine concentrations were low in this ESN population, with the following percentage of values falling below the LLD: of IL-6 20%, IL-10 72%, IP-10 17%, and RANTES 26%. *T*.*vaginalis* infection was associated with increase expression of IL-8 compared to uninfected women (1275pg/ml vs. 566 pg/ml, p = .02) and a trend toward increased expression of IL-6 (p = .07). T. vaginalis was also significantly associated with increased expression of the immune activation marker, sTNFr1 (430 pg/ml versus 264 pg/ml, p = 0.0005). There was no significant effect of *T*. *vaginalis* infection on the endocervical levels of IL-10, IP-10, or RANTES.

### Endocervical mononuclear cell analysis

**Fig 2:**
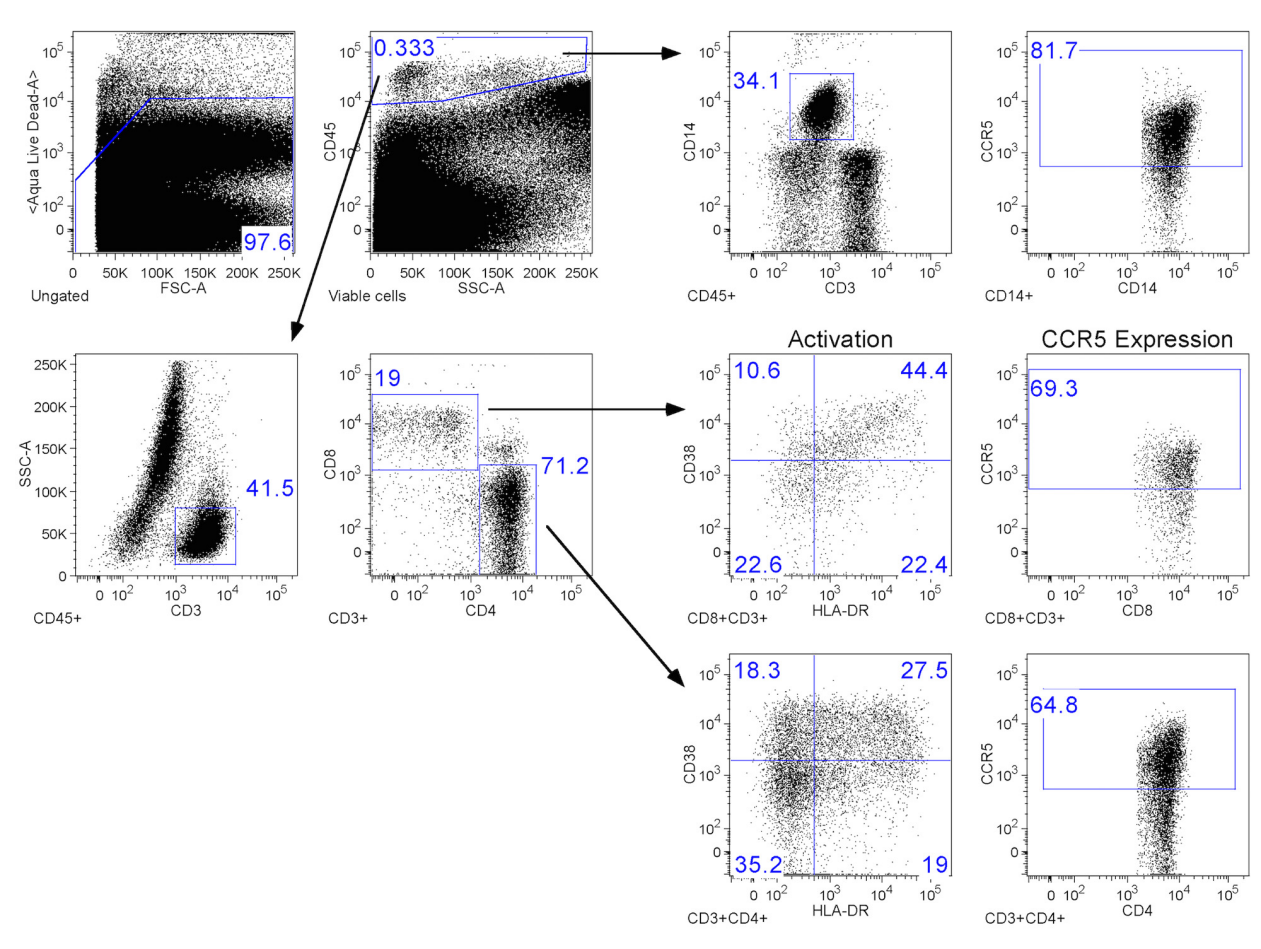
Gating strategy for mononuclear cell expression. Dead cells were excluded with a viability stain before gating on CD45+ cells. CD45+ cells were further analyzed for CD3+ and CD14+ expression. CD45+CD3+ cells were gated on CD4+ or CD8+ before HLA-DR/CD38 quadrant gating as well as CCR5 co-receptor expression. CD45+CD14+ cells were also analyzed for CCR5 expression.

Percent expression of T cell and monocyte phenotypes in the endocervix were evaluated using the gating strategy outlined in Fig 2. Cervical cytobrush samples with CD3+ event counts <200 were excluded from analysis. We also excluded an additional sample with CD3+ events >200 but with < 1% of events within the CD4+ and CD8+ gate. *T*. *vaglinalis* infection was not associated with increased expression of CCR5+ CD4+ or CD8+ cells (Table 2). It was also not associated with activated CD4+ or CD8+ cells of the HLADR+CD38+ phenotypes. In fact, we observed that *T*. *vaginalis* was associated with decreased percent expression of CD4+ cells of the CD38-HLADR+ phenotype (p = .01). Lastly, there was a trend toward decreased expression of CCR5+ monocytes in T. vaginalis infected women in the unadjusted model (p = .07). However, this relationship was no longer significant after adjusting for age and menstrual phase (p = .14).

**Table 2:** Percent expression of T cells and monocytes phenotypes in the cervix of ESN women by *T*. *vaginalis* status, N = 54.

Cell phenotype	TV-infected (n = 19)	TV-uninfected (n = 35)	UnadjustedOdds Ratio	p-value	Adjusted Odds Ratio	p-value
CD4+ T cells	49.8 (45.2, 58.7)	51.2 (42.3, 57.3)	.99	.05	.99	.06
CCR5+	74.8 (59.8, 87)	72.5 (58.5, 82.7)	1.09	.69	1.15	.52
CD38+HLADR-	11.2 (8.9, 14.5)	11.1 (6.6, 13.8)	1.09	.61	1.14	.42
CD38+HLADR+	5.8 (3.3, 11.1)	8.2 (3.6, 11.4)	.84	.39	.81	.29
CD38-HLADR+	14.2 (11.2, 22.3)	19.0 (14.7, 29.3)	.73	.03	.68	.01
CD38-HLADR-	66.0 (55.9, 73.4)	58.2 (47.3, 70.3)	1.27	.10	1.35	.03
CD8+ T cells	43.6 (32.1, 46.4)	39.4 (29.9,46.8)	1.00	.28	1.00	.32
CCR5+	81.0 (73.2, 91.6)	83.5 (70.6, 88.5)	.98	.93	1.03	.90
CD38+HLADR-	10.8 (6.9, 17.0)	14.1 (7.3, 18.5)	.82	.25	.87	.41
CD38+HLADR+	15.0 (8.7, 22.5)	14.5 (6.0, 26.3)	.94	.78	.85	.41
CD38-HLADR+	20.1 (15.8, 24.3)	19.6 (15.2, 27.6)	.99	.92	.92	.52
CD38-HLADR-	52.9 (45.7, 59.7)	49.8 (28.6 60.2)	1.23	.21	1.36	.05
CD45+CD14+ monocyte	11.6 (5.4, 19.2)	6.3 (2.5, 21.9)	1.42	.15	1.28	.30
CD14+CCR5+	78.5 (63, 86.9)	84.8 (69.6, 94.1)	.63	.07	.68	.14

Data are median percent expression (interquartile range) values. Adjusted odds ratio adjusted for age and menstrual phase. ESN = Female sex workers, TV = *Trichomonas vaginalis*.

## Discussion

Among ESN, *T*. *vaginalis* infection was associated with increased levels of the pro-inflammatory cytokine IL-8 and higher concentrations of sTNFr1 in the endocervix. However, it was not associated with increased expression of CCR5+ T cells nor activated T cells of the CD38+ and HLADR+ phenotype. While there was a trend toward increased concentrations of IL-6, *T*. *vaginalis* infection was not associated with any significant change in the concentration of the pro-inflammatory cytokines IP-10 and RANTES or the anti-inflammatory cytokine IL-10.

The overall low levels of IL-6, IL-10, IP-10, and RANTES seen in this ESN population are consistent with prior studies demonstrating immune quiescence in the genital tract and serum of this population [15, 27]. However, we demonstrate that *T*. *vaginalis* infection is associated with a reversal of this baseline immune quiescence in ESN as evidenced by increased IL-8, IL-6 and sTNFr1 expression compared to those uninfected. The positive associations between *T*. *vaginalis* infection and both IL-8 and IL-6 are consistent with previously reported studies [12, 17–19]. As previously discussed, IL-8 and IL-6 are associated with increased HIV infectivity and replication in the female genital tract [8–10]. To our knowledge, this is the first study to demonstrate an association between sTNFr1 and *T*. *vaginalis* infection. The positive association between T. vaginalis and sTNFr1 is consistent with prior *in vitro* studies demonstrating increased production of TNF-a in macrophages exposed to *T*. *vaginalis*. TNFr1 production is upregulated in response to increased TNF-a and is the pathway by which TNF-a promotes HIV viral replication [28–30]. TNFr1 promotes HIV viral replication via stimulation of nuclear factor kappa B production, which in turn induces genes involved in acute and chronic immune activation [28]. This may promote HIV infection by increasing the size of the founder population at the time of initial infection. It is unknown, however, whether it may contribute to increased susceptibility to de-novo HIV infection in exposed, seronegative individuals. Further studies are needed to determine whether increased levels of TNFr1 may directly contribute to initial HIV infection or are simply a marker of genital immune activation, which is a risk for genital HIV transmission.

While the design of this study cannot determine the exact mechanisms by which *T*. *vaginalis* might mediate an increased risk of HIV infection in ESN, the failure of *T*. *vaginalis* infection to increase percent expression of activated T cell phenotypes CD38 and HLADR suggests that T cell activation along the CD38 and HLADR pathways may not be the predominant mode by which *T*. *vaginalis* could facilitate endocervical HIV transmission. In addition, we did not see an increased percent recruitment of CCR5+ T cells in T. vaginalis infected women. Though the failure to see this relationship may have been due to the high percent expression of CCR5+ T cells in both the *T*. *vaginalis* infected and uninfected groups (81% and 83.5%; respectively). Unfortunately, we cannot comment on overall T cell recruitment because only percentages and not the absolute number of T cells were measured. It is possible that women with *T*. *vaginalis* infection had increased absolute expression of CCR5+ and activated T cell phenotypes, but in similar proportions to uninfected women. We also cannot exclude the possibility that *T*. *vaginalis* infection may stimulate increased expression of different activated T cell phenotypes such as CD69+ cells, which, as previously mentioned, has been demonstrated *in vitro* [16]. We were unable to control for other factors, such as type of contraception use and concurrent STIs that may also impact the immune profile of ESN. However, there was a low prevalence of gonorrhea and Chlamydia in our population and none of the cases occurred in the *T*. *vaginalis* infected group. Thus, concurrent STIs would not explain the significant associations seen between *T*. *vaginalis* and the immune markers discussed above. Similarly, as the predominant method of contraception used was condoms it is less likely that contraception use had a significant impact on our measurements of immune markers in the endocervix.

There may be limited generalizability of our study to other ESN populations because despite engaging in high-risk sexual behaviors (see criteria for study entry), the women in our study reported few unprotected sexual encounters prior to their study visit, with a median of one unprotected sexual encounter the week prior to the study visit and only one unprotected sexual partner in the 6 months prior. Many of these women were involved in a prior study cohort of women at risk for HIV infection, in which the median number of sexual partners decreased from a baseline of three partners in the prior 6 months to only one partner in the prior 6 months at 12 month follow-up; and this effect persisted at 18 month follow-up [31]. The reduction in sex partners was likely due to repeated safe sex education, and this modification in sexual behavior appears to have extended into this current study. It is unknown what impact a persistent reduction of sexual exposure to HIV and other STIs may have on the immune quiescent profile in these women; though this profile appears to have persisted in our ESN cohort as they had overall low levels of cytokine expression regardless of *T*. *vaginalis* infection.

In summary, we demonstrated that although *T*. *vaginalis* infection in ESN is associated with increased concentrations of IL-6, IL-8 and sTNFr1, it is not associated with a reversal of their unique immune quiescent T cell profile along the CD38 and HLADR pathways in the endocervix. These findings suggest that T cell activation along the CD38 and HLADR pathways may not be the predominant modes by which *T*. *vaginalis* could facilitate genital HIV infection. However, further studies are needed to explore whether *T*. *vaginalis* may stimulate T cell activation via alternate immune pathways, facilitating genital HIV transmission in women. Improved understanding of the mechanisms by which *T*. *vaginalis* increases genital HIV transmission are an important step toward developing novel strategies to modulate these effects and decrease sexual transmission of HIV in women.

## Supporting Information

S1 Dataset Cytokine data. (XLSX)

S2 Dataset Mononuclear cell expression data. (XLS)
